# High vs. Low Initial Oxygen to Improve the Breathing Effort of Preterm Infants at Birth: Study Protocol for a Randomized Controlled Trial

**DOI:** 10.3389/fped.2019.00179

**Published:** 2019-05-07

**Authors:** Janneke Dekker, Stuart B. Hooper, Martin Giera, Erin V. McGillick, G. Jeroen Hutten, W. Onland, Anton H. van Kaam, Arjan B. te Pas

**Affiliations:** ^1^Department of Pediatrics, Leiden University Medical Center, Leiden, Netherlands; ^2^The Ritchie Centre, Hudson Institute for Medical Research, Melbourne, VIC, Australia; ^3^Department of Obstetrics and Gynaecology, Monash University, Melbourne, VIC, Australia; ^4^Center Proteomics Metabolomics, Leiden University Medical Center, Leiden, Netherlands; ^5^Department of Neonatology, Emma Children's Hospital, Amsterdam UMC, Amsterdam, Netherlands

**Keywords:** oxygen, preterm, breathing effort, stabilization, resuscitation

## Abstract

**Background:** Although most preterm infants breathe at birth, their respiratory drive is weak and supplemental oxygen is often needed to overcome hypoxia. This could in turn lead to hyperoxia. To reduce the risk of hyperoxia, currently an initial low oxygen concentration (21–30%) is recommended during stabilization at birth, accepting the risk of a hypoxic period. However, hypoxia inhibits respiratory drive in preterm infants. Starting with a higher level of oxygen could lead to a shorter duration of hypoxia by stimulating breathing effort of preterm infants, and combined with subsequent titration based on oxygen saturation, prolonged hyperoxia might be prevented.

**Study design:** This multi-center randomized controlled trial will include 50 infants with a gestational age between 24 and 30 weeks. Eligible infants will be randomized to stabilization with an initial FiO_2_ of either 1.0 or 0.3 at birth. Hereafter, FiO_2_ will be titrated based on the oxygen saturation target range. In both groups, all other interventions during stabilization and thereafter will be similar. The primary outcome is respiratory effort in the first 5 min after birth expressed as average minute volume/kg. Secondary outcomes include inspired tidal volumes/kg, rate of rise to maximum tidal volume/kg, percentage of recruitment breaths with tidal volumes above 8 mL/kg, duration of hypoxia and hyperoxia and plasma levels of markers of oxidative stress (8-iso-prostaglandin F2α).

**Discussion:** Current resuscitation guidelines recommend oxygen titration if infants fail to achieve the 25th percentile of the SpO_2_ reference ranges. It has become clear that, using this approach, most preterm infants are at risk for hypoxia in the first 5 min after birth, which could suppress the breathing effort. In addition, for compromised preterm infants who need respiratory support at birth, higher SpO_2_ reference ranges in the first minutes after birth might be needed to prevent prolonged hypoxia. Enhancing breathing effort by achieving an adequate level of oxygenation could potentially lead to a lower incidence of intubation and mechanical ventilation in the delivery room, contributing to a lower risk on lung injury in high-risk preterm infants. Measuring 8-iso-prostaglandin F2α could lead to a reflection of the true amount of oxygen exposure in both study groups.

## Introduction

Although most preterm infants breathe at birth, their respiratory drive is weak and often insufficient to aerate their lungs and establish gas exchange (1–3). Historically, these infants were intubated and mechanically ventilated, which is associated with a higher risk of lung and brain injury (4). To minimize injury, this approach is now avoided and the focus of respiratory care has shifted to support the mechanics of breathing by providing a continuous positive airway pressure (CPAP) by face-mask (5, 6). Despite this approach, there is still a high failure rate of CPAP in preterm infants in case of a weak respiratory drive, associated with higher mortality and major morbidities (7). It has become clear that the best way to facilitate air entry into the lungs using non-invasive respiratory support is to stimulate spontaneous breathing (8, 9), which is also likely to be the most gentle and effective way of providing respiratory care without causing injury. Therefore, the focus of respiratory care should include both support of the mechanics of breathing and stimulation of spontaneous breathing. Currently, international guidelines recommend tactile stimulation and some local guidelines additionally advocate the use of caffeine in the delivery room to stimulate breathing in addition to the use of non-invasive ventilation (5, 10, 11).

Even with these techniques to support and stimulate spontaneous breathing, achieving adequate oxygenation with a low initial fraction of inspired oxygen (FiO_2_) during stabilization has been shown to be difficult (12, 13). Most preterm infants born with a gestational age below 32 weeks are therefore at risk for hypoxia (14). This, in turn leads to inhibition of respiratory drive because the inhibitory effect of hypoxia on fetal breathing movements persists well-into newborn life (2). Increasing the FiO_2_ can reduce the level of hypoxia leading to an increase of respiratory effort in preterm infants. This was demonstrated in an observational study where an increase in respiratory drive was observed after switching FiO_2_ from 0.21 to 1.0 at some point during stabilization at birth (15).

The optimal initial oxygen concentration for stabilizing infants after birth is still a subject of debate, as both hypoxia and hyperoxia can cause damage of multiple organ systems (16, 17). Hypoxia may lead to delayed cellular damage, (18) as during hypoxia the production of free radicals will be provoked, by an elevated level of hypoxanthine (19). On the other hand, high oxygen levels increase the biosynthesis of prostaglandins which are an additional mechanism inhibiting chemoreceptors in the respiratory center (20). In addition, free radical production (associated with both oxidative and nitrosative stress) increases during hyperoxia, which can overwhelm the relatively immature antioxidant capacity of the preterm infant. The resulting excess of free radicals cause wide-spread damage to cells, enzymes, lipids, DNA, and proteins (21–24).

The recent meta-analysis of Oei et al. showed a higher mortality rate in the group of preterm infants (< 32 weeks of gestation) resuscitated with an initial FiO_2_ of 0.21 compared to the group resuscitated with an initial FiO_2_ of 1.0 (18). Furthermore, when stabilization of preterm infants after birth was started with low oxygen concentrations (FiO_2_ 0.21–0.3), oxygen saturation (SpO_2_) and heart rates increased more slowly than the SpO_2_ references outlined in the international guidelines, despite the use of respiratory support. This indicates that when starting respiratory support with lower oxygen concentrations preterm infants are at a higher risk for hypoxia (25). Indeed, this was shown in the study of Goos et al. (12) and White et al. (13).

Regardless of the initial FiO_2_ level, in all studies most infants ended up with a FiO_2_ between 0.4 and 0.6 after 5 min to reach SpO_2_ target ranges. Currently, to prevent hyperoxia and achieve normoxia in newborns, international guidelines recommend to start stabilization after birth in preterm infants with low FiO_2_ (0.21–0.3) (26), after which FiO_2_ should be titrated based on SpO_2_ target ranges (16, 17, 27). In this recommendation, a period of low oxygen saturation is accepted and the effect of hypoxia on respiratory effort seems to be disregarded (28).

We hypothesize that with an initial FiO_2_ of 1.0, infants will reach SpO_2_ target ranges more rapidly, leading to less hypoxia and an increase in respiratory effort. With careful titration of FiO_2_ based on SpO_2_ target ranges, we hypothesize that the occurrence of hyperoxia will be minimized.

The aim of this current study is to determine the effect of an initial FiO_2_ of 1.0 compared to standard management of an initial FiO_2_ of 0.3 on breathing effort of preterm infants immediately after birth.

## Study Design and Population

### Study Design

The design of this multi-center study is a randomized clinical trial. Eligible infants between 24^+0^ and 29^+6^ weeks of gestation will be randomized to either start stabilization with an initial FiO_2_ of 1.0 or 0.3.

### Study Population

The study will take place at the NICU of the Leiden University Medical Center (LUMC) and the NICU of the Amsterdam UMC (AUMC), both located in the Netherlands. Infants with a gestational age between 24^+0^ and 29^+6^ weeks, born at the LUMC and the AMC will be eligible for inclusion. Infants with a congenital abnormality or condition that might have an adverse effect on breathing or ventilation will be excluded, such as congenital diaphragmatic hernia, trachea-esophageal fistula or cyanotic heart disease.

### Primary Outcome

The primary outcome is respiratory effort, expressed as average minute volume normalized for body weight in the first 5 min after birth.

### Secondary Outcomes

Secondary outcomes include;

- Average tidal volumes in the first 5 min after birth
- Average rate of rise to maximum tidal volumes in the first 5 min after birth
- Percentage of recruitment breaths with tidal volumes above 8 mL in the first 5 min after birth
- Duration of hypoxia (defined as oxygen saturation < 25th percentile of the target ranges defined by Dawson et al.) in the first 10 min after birth
- Duration of hyperoxia (defined as oxygen saturation > 95%) in the first 10 min after birth
- Changes in diaphragmatic activity in the first 5 min after birth
- Incidence of intubation in the delivery room
- Concentration of 8-iso-prostaglandin F2α (29), an oxidative stress metabolite associated with hyperoxia in blood.

Volumes measured will be normalized for body weight. In addition, the interaction between those primary and secondary outcomes and the time after birth will be assessed.

### Other Outcomes

Other outcomes that will be collected include;

- Percentage of time of facemask ventilation applied during stabilization at birth
- Average oxygen saturation and heart rate in the first 5 min after birth
- Oxygen saturation and heart rate *per minute* in the first 5 min after birth
- Percentage of spontaneous breaths with tidal volumes above 4 mL/kg
- Percentage of time that oxygen saturation is within 90–95% range in the first 5 min after birth
- Percentage of time that oxygen saturation is within 90–95% range between 5 and 10 min after birth
- Percentage of time that oxygen saturation is within target ranges defined by Dawson et al. (27) in the first 10 min after birth.

In addition, we will collect basic characteristics such as gestational age, birth weight, mode of delivery, incidence of complications in pregnancy, use of antenatal corticosteroids, maternal medication use, and Apgar score 1 and 5 min after birth.

This study is not designed to demonstrate any differences in clinical short- and long-term outcomes. However, we will record study parameters focusing on short-term clinical outcomes, such as incidence of intubation in the delivery room and in the first 24 h after birth, pneumothorax, intraventricular hemorrhages (IVHs) grade 3 or more and neonatal death.

### Sample Size Calculation

There is no data available on measurements of respiratory effort in the first 5 min after birth during stabilization with a FiO_2_ of 1.0 compared to 0.3. In a recent study, the average minute volume of preterm infants over the first 100 breaths was 150 ± 70 mL/kg/min (30). In a retrospective observational study, we observed an 80% relative increase in respiratory drive when FiO_2_ was switched from 0.21 to 1.0, in the first minute after FiO_2_ was increased (from 134 to 240 mL/kg/min). Other parameters of respiratory effort increased as well: average expired tidal volumes (Vt_e_) increased by 37% (from 4.9 to 6.7 mL/kg), and rate of rise to maximum tidal volumes increased by 32% (13.8 to 18.2 mL/kg/s) (15).

However, since we investigate a FiO_2_ of 0.3 instead of 0.21 in the lower oxygen arm, we expect half of the effect (40% relative increase) in respiratory drive than demonstrated in the study of Van Vonderen et al. (15). Therefore, to detect an increase in average minute volume in the first 5 min after birth from 150 to 210 mL/kg/min, using a standard deviation of 70 mL/kg/min, with a power of 80% and an α error of 5% (two tailed test), 22 infants are required for each arm. Given the fact that parents may withdraw their consent or decline deferred consent after the infant is included in the study, or that the recording of physiological measurements fails in 10% of all infants, a total of 50 infants will be recruited (25 in each arm).

## Treatment of Subjects

### Randomization

Allocation will be stratified by gestational age. Infants of 24^+0^-26^+6^ weeks and 27^+0^-29^+6^ weeks will be included using variable block (4–6) sizes. Concealment of allocation will be ensured by using the randomization process of Castor EDC (Amsterdam, The Netherlands), an electronic data capture system. The neonatal fellow or neonatal consultant in charge of the procedure will randomize using Castor EDC.

### Study Procedures

Infants will be randomized to start stabilization after birth with an initial FiO_2_ of either 1.0 or 0.3, after which FiO_2_ will be titrated following target SpO_2_ recommended in our local guidelines on stabilization after birth, which are based on the target ranges described by Dawson et al. (27). In both groups, all other procedures in the delivery room and NICU will be performed according to the local and international guidelines, only the initial FiO_2_ setting will be different. Cord clamping will be performed after 30 s in case of an apneic infant, and after 60 s if the infant is breathing at birth.

After the initial FiO_2_ setting, titration will be performed according to the local protocol:

-When SpO_2_ is below target ranges defined by Dawson et al. FiO_2_ will be titrated up to 0.5 and subsequently to 1.0, when the initial FiO_2_ setting was 0.3. In case of an initial FiO_2_ setting of 1.0, FiO_2_ will be maintained at 1.0.
-When SpO_2_ is above target ranges, FiO_2_ will be titrated down to 0.5 and subsequently to 0.3 and 0.21. If SpO_2_ is above target ranges when stabilization is initiated with FiO_2_ 0.3, FiO_2_ will be titrated down to 0.21 directly.

#### Measurements

##### Measurement of respiratory function

In both groups, standard care will be provided in the delivery room and NICU and local guidelines on stabilization after birth will be followed, using a Neopuff^TM^ infant T-piece resuscitator (Fisher & Paykel Healthcare, Auckland, New Zealand) or a Giraffe Star System (Anandic Medical Systems, Feuerthalen, Switzerland). Respiratory function monitoring at birth will be recorded during stabilization in the first 10 min of life, which is standard practice in our units. The respiratory function monitor will be used to measure inflation pressures, flow and tidal volumes. This can be used to measure average minute volume, average rate of rise to maximum tidal volumes, and the total time of ventilation given. It uses a small variable resistance anemometer to measure gas flow in and out of a facemask or endotracheal tube. This signal is automatically integrated to provide inspired and expired tidal volumes. The difference equals the leak from the facemask or endotracheal tube.

Breathing effort will also be measured by activity of the diaphragm using electromyography (EMG) with the Dipha-16 (Demcon, Groningen, The Netherlands). The Dipha-16 is used to record spontaneous breathing (EMG of diaphragm) and heart rate (ECG). Both parameters are used for audit of stabilization, which is standard care in the NICU of the LUMC. Three EMG electrodes connected to the Dipha amplifier (similar to ECG electrodes) will be placed. Two are placed bilateral at costo-abdominal margin in the nipple line, and the common electrode will be placed on one of the legs.

To record SpO_2_ and heart rate a Masimo SET pulse oximeter probe (Masimo Radical, Masimo Corporation, Irvine, California, USA) or Nellcor^TM^ pulse oximeter probe (Covidien, Dublin, Ireland) is placed around the right wrist or hand of the infant. The FiO_2_ is measured using a portable oxygen analyzer AX300-I (Teledyne Analytical Instruments, CA, USA), and the airway pressures are registered by a variable orifice flow sensor (Avea Varflex Flow Transducer, Carefusion, Yorba Linda, CA, USA) connected to the facemask measuring the flow in and out the infant. The signals are digitized at 200 Hz using the NewLifeBox-R physiological recording system (Advanced Life Diagnostics, Weener, Germany) and all signals are recorded by the NewLifeBox Neo-RSD computer system (Advanced Life Diagnostics, Weener, Germany) supported by Polybench physiological software (Applied Biosignals, Weener, Germany). Pulmochart software (Applied Biosignals, Weener, Germany) is used to analyze primary and secondary outcomes.

##### Measurement of metabolites of oxidative stress in blood

Chemical oxidation products of polyunsaturated fatty acids, formed by peroxidation initiated by free radicals, are shown to be the best biomarkers to assess oxidative stress in humans. Of oxidized fatty acids, the best indicator for oxidative stress is 8-iso-prostaglandin F2α (31). Cord blood 8-iso-prostaglandin F2α is a stable marker of lipid peroxidation at birth, and will therefore be analyzed as baseline value for oxidative stress obtained after birth (32).

Measurement time points:

- Cord blood will be drawn directly after birth as a baseline value.
- At ~1 h after birth, a blood sample will be taken together with the sample normally taken for measuring standard analyses; this ensures that the infant is not exposed to an extra venepuncture or capillary puncture. The total amount of inspired oxygen given from birth will be recorded from birth until the sample is taken.
- At 24 h after birth, again together with measurement of standard blood analyses.

For every sample, a total of 300 μL blood will be used to measure 8-iso-PGF2α. A preterm infant of 500 gram has ~40 mL of total blood volume, whereas an infant of 1,000 gram has ~80 mL. As only 2 samples will be taken from the preterm infants, only 600 μL of blood will be withdrawn, which accounts for 0.75–1.5% of total blood volume.

## Procedures

### Recruitment and Consent

In addition to the initial FiO_2_ setting, standard care is provided in both groups. Respiratory data is collected as standard clinical care. As the starting FiO_2_ is randomly assigned, we will ask for parental consent. Antenatal consent will be obtained before birth if the mother is hospitalized in the LUMC or AUMC with imminent preterm labor before 30 weeks of gestation. Whenever antenatal consent is obtained, but the mother passes the term of 30 weeks of gestation, the infant will not be included in this study.

In case the condition of the mother and/or fetus suddenly deteriorates, resulting in an emergency situation with immediate delivery, it may not be possible to ask for antenatal consent from either parent. Also, when the mother arrives in hospital in full labor and tocolysis is not an option, it may not be possible, or appropriate to ask for antenatal consent. Emergency situations after birth cannot always be predicted and the neonatologist may have to act upon the infant's interest without being able to discuss the treatment plan with the parents, let alone ask for study consent. The worsening of the condition of these infants, who are often also not pre-treated with antenatal steroids, will increase the chances for respiratory insufficiency after birth and the need for respiratory support in the delivery room. Exclusion of this group of infants will therefore cause an important selection bias.

For the infants born in the following situations, retrospective consent will be used:

1. In case of an emergency situation (e.g., the mother is in full labor or immediate delivery is necessary), when there is not enough time for antenatal consent.
2. When obtaining antenatal consent is inappropriate (e.g., the condition of the mother does not allow for proper consideration of both parents on whether or not to participate in a trial). In this case there is enough time, though trying to obtain informed consent in this situation would be inappropriate.

This study fulfills the criteria for a waiver of informed consent in Europe (Human Medicines Evaluation Unit 1995), Canada (Natural Sciences and Engineering Research Council of Canada 2006) and the United States (USA Food and Drug Administration 1999) and Australian NHMRC guidelines for studies in emergency medicine (National Health and Medical Research Council 2002). For these infants, retrospective consent will be sought from the parents to use data obtained. For this important study to be feasible, and enroll a representative sample, a waiver of informed consent is appropriate when consent cannot be obtained prior to delivery.

The parents will be informed as soon as possible after the study and asked to consent to data being collected on their infant. There are many precedents of randomized trials conducted with such a consent waiver in emergency situations in neonatal stabilization after birth and adult resuscitation.

While undertaking research studies without prior informed consent needs careful consideration, studies of emergency medical interventions, such as neonatal stabilization after birth, are exceptionally difficult if prior consent is required. Arguably, it is more unethical to use treatments that have not been studied than to evaluate them in a carefully controlled manner (23, 33).

### Withdrawal of Individual Subjects

Parents can withdraw their consent at any time for any reason if they wish to do so without any consequences. When parents want to withdraw their infant for any reason, this infant will be handled as drop-out. As we included an additional 10% of infants in the sample size calculation, this will not influence the power calculation.

### (Serious) Adverse Events

Adverse events are defined as any undesirable experience occurring to a subject during the study, considered related to the initial FiO_2_ setting of 1.0. All adverse events observed by the investigator or the staff will be recorded and noted to the medical ethical committee of the LUMC.

This study population (preterm infants) has a high risk of serious complications, which are inherent to their vulnerable condition and unrelated to the intervention that is under evaluation in this trial. These complications (pneumothorax, bronchopulmonary dysplasia (BPD), retinopathy of prematurity (ROP), IVHs grade 3 or more, cystic periventricular leukomalacia (PVL), necrotizing enterocolitis (NEC) grade 2 or more, death) are not expected to be related to the intervention (starting stabilization after birth with a FiO_2_ of 1.0 vs. 0.3) as the intervention will be performed only at the start of stabilization, after which in both groups titration will be performed. In light of the above, immediate and individual reporting of all these conditions related complications will not enhance the safety of the study, and therefore these complications will be handled as adverse events.

### Statistical Analysis

Data will be presented as median [interquartile range (IQR)], mean ± standard deviation (SD) or number (percentage) where appropriate. Data will be checked for meeting the assumption of normality first by making a frequency distribution per minute. Outliers will be identified and handled according to the data (outliers will be deleted if there are arguments to do so, or transformed if not). Intention-to-treat will be employed. Statistical analysis will be performed with SPSS software version 23.0 (SPSS, Chicago, Illinois; 2012).

#### Statistical Analysis of Primary Outcome

Differences in average respiratory minute volume in the first 5 min after birth between the study groups will be tested with a Student's *t*-test if the data are found to be parametric, or with Mann-Whitney u-test when the data are non-parametric.

#### Statistical Analysis of Secondary Outcomes

- The interaction of respiratory minute volume, tidal volumes, rate of rise to maximum tidal volumes and diaphragm activity in the first 5 min after birth with time will be analyzed with a linear mixed model, in which both study group (treatment) and time will be taken in consideration.
- Differences in average tidal volumes, average rate of rise to maximum tidal volumes, and percentage of recruitment breaths will be tested with a Student's *t*-test if the data is found to be parametric, or with Mann-Whitney *u*-test when the data is non-parametric.
- The concentration of 8-iso-prostaglandin F2α will be analyzed with a linear mixed model, in which both study group (initial FiO_2_ 0.3 vs. 1.0) and time will be taken in consideration.

#### Statistical Analysis of Other Outcomes

Other study parameters and basic characteristics will only be explored to test for differences between the study groups. If there are found to be significant differences with clinical relevance for the primary outcome, this will be taken into account when analyzing the data by incorporating these differences in the linear mixed model.

### Ethical Considerations

The study will be conducted according to the principles of the Declaration of Helsinki and in accordance with the Medical Research Involving Human Subjects Act (WMO). The study protocol is approved by the ethical committee of the LUMC and AUMC.

### Dissemination of Results

Results of the study will contribute to a manuscript, which will be submitted for publication to a peer-reviewed international medical journal, and will be presented at national and international conferences.

## Discussion

Most preterm infants need additional oxygen during stabilization at birth to achieve appropriate SpO_2_ target ranges (34). Results of a recent meta-analysis demonstrated that persisting hypoxia at 5 min after birth is associated with a higher risk of mortality and the development of an intraventricular hemorrhage, which we should try to avoid (14). Although a recent systematic review showed no differences in percentage of infants achieving SpO_2_ target ranges between infants initiating stabilization with high or low FiO_2_, the level of evidence of these results is low (35). It is therefore still unclear whether the initial FiO_2_ level influences breathing effort, thereby influencing the capacity of preterm infants to reach the target range. However, it has recently became clear that oxygenation can only be influenced in infants supported by non-invasive ventilation in case of presence of spontaneous breathing, as then the larynx opens and an open airway is achieved (9). Before focusing on achievement of SpO_2_ target ranges, we should look at the effect of initiation stabilization with a high FiO_2_ on breathing effort.

The current SpO_2_ target ranges used during stabilization at birth are based on the reference values described by Dawson et al. (27). While these target ranges have been based on data from healthy term and preterm infants, those values might not apply to infants requiring more respiratory assistance at birth. In the current resuscitation guidelines, oxygen titration is recommended if infants fail to achieve the 25th centile of the SpO_2_ reference ranges and are thus hypoxemic in the first minutes after birth (14, 36). Because hypoxia is known to provide inhibitory input into the respiratory center, this effect on respiratory effort has been overlooked with regard to the importance of oxygen titration. It is possible that the compromised preterm infant requiring respiratory support during transition would benefit by higher SpO_2_ reference ranges during stabilization at birth. By initiating stabilization at birth with a FiO_2_ of 1.0, we hypothesize that infants will achieve higher SpO_2_ values in the first minutes after birth, leading to an increase in respiratory effort at birth.

A FiO_2_ of 1.0 should be administered with caution to avoid the detrimental effects of hyperoxia (21, 23, 24). However, in this study the amount of time that infants receive pure oxygen is very limited since FiO_2_ will be titrated according to SpO_2_ values. To evaluate pure oxygen exposure, both the amount of time that infants spent above SpO_2_ target range and the level of 8-iso-prostaglandin F2α indicating oxidative stress will be measured.

While low SpO_2_ values reflect a low oxygenation level in arterial blood, known as hypoxaemia, this does not necessarily represent low oxygen uptake and usage by tissues, particularly cerebral tissues. While pO_2_ gradients drive oxygen diffusion into tissues and pO_2_ levels in blood are related to blood SpO_2_ levels, there are any factors that determine oxygen uptake and usage by tissues. For instance, organs can increase blood flow to maintain oxygen delivery and/or increase oxygen extraction when blood oxygen content decreases. As such, SpO_2_ alone provides limited information on oxygen consumption by tissues at high or low blood oxygen levels. In addition, individual patients might differ from one another in their ability to cope with free radicals released in response to inflammation, hyperoxia, and hypoxia before or after birth (21). Although the study is not powered to detect a difference in oxidative stress, we decided to objectively measure this using 8-iso-prostaglandin F2α as marker of oxidative stress, which indicates the true amount of oxygen exposure.

The reported trials comparing high vs. low levels of oxygen did not report on how oxygenation levels influence respiratory effort. Although the level of respiratory support during stabilization at birth was reported in most trials and shown to be not significantly different, the effectiveness of spontaneous breathing in both infants receiving higher or lower FiO_2_ was not evaluated (18, 37–40). Gaining an adequate level of spontaneous breathing during stabilization at birth to improve the success of non-invasive respiratory support could potentially lead to a lower incidence of intubation and mechanical ventilation in the delivery room, contributing to a lower risk of lung injury in high-risk preterm infants.

## Ethics Statement

The ethical committee of the LUMC and AUMC approved the study protocol. Informed parental consent was obtained antenatally when possible. In case of an emergency situation (e.g., mother in full labor or when immediate delivery was necessary) or when obtaining antenatal consent was inappropriate (e.g., if the condition of the mother did not allow for proper consideration on participation), consent was asked retrospectively. This study was registered in www.trialregister.nl, with registration number NTR6878.

## Author Contributions

JD, MG, EM, GH, and AtP conceptualized the study. JD wrote the first draft of the manuscript. JD, SH, MG, EM, GH, WO, AvK, and AtP contributed to interpretation of the study protocol in relation to other studies performed on this subject (discussion). All the authors critically reviewed and contributed to the final draft of the manuscript.

### Conflict of Interest Statement

The authors declare that the research was conducted in the absence of any commercial or financial relationships that could be construed as a potential conflict of interest.
